# Chromosome-Scale Genome Assembly and Genome-Wide Identification of Antimicrobial Peptide-Containing Genes in the Endangered Long-Finned Gudgeon Fish (*Rhinogobio ventralis*)

**DOI:** 10.3390/biology14111486

**Published:** 2025-10-24

**Authors:** Jieming Chen, Xinhui Zhang, Yanping Li, Yunyun Lv, Xinxin You, Qiong Shi, Zhengyong Wen

**Affiliations:** 1 Key Laboratory of Sichuan Province for Fishes Conservation and Utilization in the Upper Reaches of the Yangtze River, Neijiang Normal University, Neijiang 641100, China; chen_jm02@163.com (J.C.); zhangxhui1987@163.com (X.Z.); liyanping@njtc.edu.cn (Y.L.); lvyunyun@njtc.edu.cn (Y.L.); 2 Shenzhen Key Lab of Marine Genomics, BGI Academy of Marine Sciences, Shenzhen 518081, China; yxx2004449@163.com; 3 Laboratory of Aquatic Genomics, College of Life Sciences and Oceanography, Shenzhen University, Shenzhen 518057, China; 4 College of Fisheries, Neijiang Normal University, Neijiang 641100, China; 5 College of Life Sciences, Neijiang Normal University, Neijiang 641100, China

**Keywords:** *Rhinogobio ventrali*, whole-genome sequencing, chromosome construction, antimicrobial peptide (AMP), AMP-containing gene

## Abstract

**Simple Summary:**

Long-finned gudgeon fish, *Rhinogobio ventralis*, is an economically important cyprinid species with a native distribution in the upper tributaries of the Yangtze River, China. Its natural population has significantly declined due to overfishing and habitat destruction in recent decades, and it is therefore classified as endangered in China. Despite its ecological and economic importance, the lack of genomic resources has restricted comprehensive studies in various areas including population conservation, ecological adaptation, and aquaculture development. To resolve this limitation, we established a high-quality chromosome-level genome assembly of *Rhinogobio ventralis* in this study. Furthermore, genome-wide prediction and localization of antimicrobial peptides (AMPs) containing genes were performed using some reference sequences from public databases. Our findings hence provide a valuable genetic resource for understanding innate immunity and developing novel bioactive compounds.

**Abstract:**

As an economically important species endemic to the upper tributaries of Yangtze River in China, long-finned gudgeon fish (*Rhinogobio ventralis*) has been classified as endangered due to habitat destruction and population decline. In this study, we constructed a chromosome-level genome assembly of *R. ventralis* by integration of MGI, PacBio and Hi-C sequencing technologies. The final genome assembly was 1015.9 Mb in length (contig N50: 25.91 Mb; scaffold N50: 39.99 Mb), and 97.19% of the haplotypic genome sequences were anchored onto 25 chromosomes. Repetitive elements accounted for 51.00% of the entire genome assembly. A total of 23,220 protein-coding genes were predicted for the assembled genome, of which 99.79% were functionally annotated. Genome evaluation revealed 99.72% completeness for the genome assembly. Through genome-wide prediction of antimicrobial peptides (AMPs), we identified and localized 561 putative AMP-containing genes in the *R. ventralis* genome. These genes were further classified into 185 distinct functional categories based on public databases, with the top ten components of Penetratin (21.74%), Histone (5.70%), E6AP (4.09%), Scolopendin 1 (2.67%), D38 (2.31%), WBp-1 (2.13%), Defensin (2.13%), Claudin 1 (1.96%), Azurocidin (AZU1, 1.78%), and Ubiquitin (1.60%). Our data presented here provide a potential genetic resource for promoting fundamental research and wild population conservation of this endangered fish species.

## 1. Introduction

The Yangtze River is the longest river in China, and it traverses varied geological terrains with over 40 tributaries, sustaining a high biodiversity of aquatic and riparian species. In the 1990s, China initiated a fundamental construction project of Three Gorges Reservoir (TGR) in the upper Yangtze River. After completion of the Three Gorges Dam, the subsequent impoundment of the TGR transformed upper region ecosystems from a lotic (flowing-water) into a lentic (still-water) environment. This alteration significantly affected aquatic animals inhabiting both upstream and downstream of the dam [1,2]. Prior to 2017, a total of 443 fish species (including 194 endemic taxa) were recorded in the Yangtze River, but this number declined to 323 (109 endemic taxa) by 2021 [3]. Scientific studies revealed that lotic-adapted and insectivore fish populations dramatically declined in many areas closed to the TGR [4].

Genus *Rhinogobio*, belonging to the family Cyprinidae and the order Cypriniformes, contains two endangered species (*R. ventralis* and *R. cylindricus*) that inhabit the upper basins of the Yangtze River [5]. *R. ventralis* (long-finned gudgeon fish) is a benthic insectivore fish and inhabits high-flow streams of the upper tributaries of Yangtze River [5,6]. Following the first and second impoundment phases of the TGR, *R. ventralis* and other lotic-dependent fish species were presumed to have migrated away from the reservoir’s vicinity [1,7]. However, after the final impoundment in 2011, the natural population of *R. ventralis* experienced a sharp decline process [7]. In 2016, it was designated as a second-class protected aquatic wildlife species in China. Beyond its traditional importance as a local economic fish, *R. ventralis* also represents a promising model species for studying lotic-adaptation and endemic fish conservation [8]. Captive breeding is becoming a vital conservation tool for this endangered fish species. To date, previous studies have demonstrated that *R. ventralis* is susceptible to infection of certain pathogens, such as *Aeromonas veronii* [9] and *Ichthyophthirius multifiliis* [10] that are two major disease-causing agents in aquaculture practices.

Antimicrobial peptides (AMPs) are a class of short cationic polypeptides induced by pathogens infection, ultraviolet radiation, temperature or other environmental stresses, exhibiting broad-spectrum antimicrobial activity and playing significant immunomodulatory roles [11]. Natural AMPs originate either from specialized AMP genes (including piscidins, defensins, and cathelicidins) or are produced through proteolytic cleavage of proteins encoded by AMP-containing genes (such as histone, NK-lysin, and chemokine genes) [12]. In practical aquaculture, development of resistance to conventional antibiotics had caused a major economic loss, which led to considerable attention to the potential applications of AMPs. The immunomodulatory functions of various AMPs have drawn much more attention, especially from AMP-containing genes in recent years. Some AMPs from teleost also show potential application for drug development. For example, grass carp IFN1 derived AMPs were reported to have antimicrobial and anti-inflammatory effects in mammals [13].

Genome-wide screening is an effective high-throughput method to identify putative AMPs. With the availability of high-quality genome assemblies, numerous putative AMP sequences were successfully predicted and validated in various fishes, such as giant grouper [14], lined seahorse [15], amphibious mudskippers [16], and black rockfish [17]. The genome-wide prediction enables systematic characterization of AMP-containing genes in target species, revealing their abundance, diversity, and genomic organization, which are essential for investigation of antibacterial mechanisms, immune modulation, host-pathogen coevolutionary adaptation, as well as drug development.

In recent years, massive amounts of genome assemblies are available in public databases because of the rapid development of sequencing technologies with low cost. As a result, we have previously reported several chromosome-level genome assemblies of endemic fish distributed in the upper basins of Yangtze River, including elongate loach (*Leptobotia elongata*) [18], Lixian plateau loach (*Triplophysa lixianensis*) [19] and wide-bodied sand loach (*Sinibotia reevesae*) [20]. *R. ventralis* possesses significantly ecological and economic significance, but it remains poorly studied due to lack of valuable genomic data. In our present study, we obtained a chromosome-level genome assembly for *R. ventralis* by integration of MGI, PacBio and Hi-C sequencing technologies. Meanwhile, we conducted genome-wide prediction and localization of putative AMP-containing genes in the assembled genome. Our data presented in this study provide a potentially valuable genetic resource for in-depth studies on ecological adaptation, evolution, and population conservation of this endemic fish species.

## 2. Materials and Methods

### 2.1. Sample Collection

**Figure 1:**
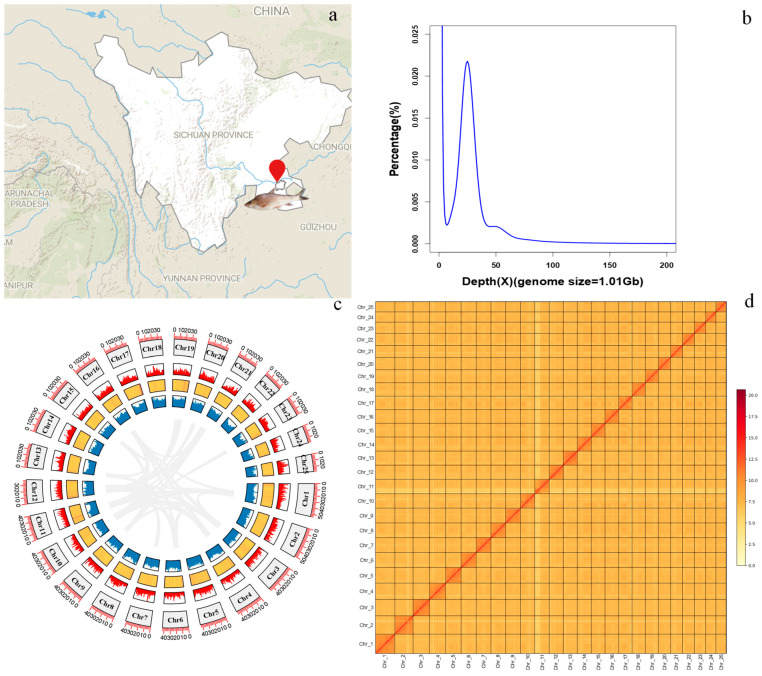
Chromosome-level genome assembly of *R. ventralis*. (**a**) Geographic map of the sample collection site (indicated by a red symbol). (**b**) K_mer (17-mer) distribution for the genome-size estimation. (**c**) Genomic features of the 25 chromosomes. From the outer to the inner rings include chromosomes, gene distribution, GC content (a yellow-to-red color gradient represents increasing GC-content), repeat elements, and collinear blocks among each chromosome. (**d**) Genome-wide chromatin interactions at a 1000-kb resolution in the 25 chromosomes. Color blocks represent the interactions, with various strengths from yellow (low) to red (high).

An adult female *R. ventralis* (body length: 244.3 mm, body weight: 152.7 g) was collected from the upper Yangtze River mainstem in Dadukou District (28°44′20.141″ N, 105°14′20.040″ E; Figure 1a) of Luzhou City, Sichuan Province, China. Tissue samples were stored at −80 °C for subsequent DNA and RNA extraction.

Genomic DNA (gDNA) was extracted from muscle using QIAamp DNA Mini kit (Qiagen, Valencia, CA, USA), and then quality and quantity of the gDNA were assessed through agarose gel electrophoresis and an Agilent 2100 Bioanalyzer (Agilent Technologies, Palo Alto, CA, USA). Total RNA samples were separately isolated from eight tissues (brain, eye, gill, muscle, heart, intestine, kidney, and liver) using TRIzol reagent (TIANGEN, Shanghai, China), and DNA contamination was eliminated with Qiagen RNeasy Mini Kits (Qiagen, Germantown, MD, USA).

### 2.2. Library Construction and Whole-Genome or Transcriptome Sequencing

MGI libraries with an insert size of 350 bp were constructed by using MGIEasy Universal DNA Library Preparation Kit (MGI, Shenzhen, China), and then they were sequenced on a DNBSEQ T7 platform (MGI, Shenzhen, China). To obtain PacBio HiFi long-reads, SMRT bell libraries (insert size of 15 kb) were generated and sequenced on a PacBio Sequel II platform in accordance with PacBio’s standard protocol (Pacific Biosciences, Menlo Park, CA, USA). For Hi-C sequencing, a Hi-C library was prepared under the manufacturer’s standard experimental guidance (GrandOmics, Wuhan, China) and then sequenced on a DNBSEQ T7 platform (MGI).

For the transcriptome sequencing of eight tissues (brain, eye, gill, muscle, heart, intestine, kidney, and liver), total RNA was used for construction of IIlumina cDNA libraries followed the manufacture’s protocol, which were subsequently sequenced on a HiSeq X Ten platform (Illumina, San Diego, CA, USA). Around 6~10 Gb of raw data were generated for assistance to gene annotation.

### 2.3. Genome Assembly and Evaluation

To estimate the genome size of *R. ventralis*, we performed a traditional 17-mer frequency distribution analysis using high-quality MGI short reads, according to the following formula: genome size = K_num/K_dept [21]. Genome-size estimation was conducted by utilizing KMC v3.0.3 and GCE v1.0.2 software [22].

*De novo* genome assembly of *R. ventralis* was constructed using Hifiasm (v0.16.0) [23] with default parameters and the high-quality HiFi long-read sequencing data. Subsequently, filtered Hi-C reads were aligned onto the initial assembly via Bowtie2 v2.2.5 [24] with default parameters. HiC-Pro v3 [25] pipeline with default parameters was employed to detect valid contact paired reads. Next, the chromosome-scale assembly was generated by using YaHS v1.2.2 [26] with default parameters, followed by manual curation with Juicebox v1.11.08 [27]. Finally, the Hi-C genome assembly was subjected to TGS-GapCloser v1.2.1 [28] with default parameters to fill N-gap with HiFi reads.

Genomic completeness was comprehensively evaluated using three complementary approaches with default parameters, including (1) CRAQ v1.0.9 [29] for systematic error assessment, (2) Merqury v1.3 [30] for k-mer-based analysis, and (3) Compleasm v0.2.6 [31] for alignment analysis with the actinopterygii_odb10 database as the reference.

### 2.4. Gene Prediction and Annotation

Homology-based and *de novo* prediction methods were employed to identify repeat elements (REs) in the *R. ventralis* genome assembly. For the homology-based prediction, RepeatMasker v4.0.6 [32] and RepeatProteinMask v4.0.6 [32] were applied to predict TEs. Meanwhile, LTR_FINDER v1.0.6 [33] and RepeatModeler v1.0.8 [34] were conducted to build a repeat library for *de novo* prediction, respectively. Then RepeatMasker v4.0.6 [32] were used to integrate the two libraries to identify TEs against the assembled genome.

To predict protein-coding genes, we implemented three strategies incorporating *de novo* prediction, homology-based method, and transcriptome-supported annotation. AUGUSTUS v3.2.1 [35] was applied for the ab initio gene prediction. Meanwhile, GeMoMa v1.6.4 [36] was conducted to perform homology prediction with genome annotation of five fish species as queries, including common carp (*Cyprinus carpio*), zebrafish (*Danio rerio*), medaka (*Oryzias latipes*), golden-line barbel (*Sinocyclocheilus anshuiensi*) and rohu (*Labeo rohita*). Additionally, transcriptome data from eight tissues (Table 1) were mapped on the genome assembly via Trinity v2.5.13 [37]. Finally, Evidence Modeler (EVM) pipeline v1.04 [38] and PASA v2.3.3 [38] software were employed for integrating the three sets of predicted genes.

Finally, we conducted function assignments for all predicted genes by using BLASTP v 2.2.26 (e-value 1 × 10^−5^) against five public databases, including SwissProt, Kyoto Encyclopedia of Genes and Genomes (KEGG), EuKaryotic Orthologous Groups (KOG), Gene Ontology (GO) and NCBI Non-Redundant Protein Sequence (NR).

### 2.5. Genome Comparison and Genomic Synteny

To evaluate the quality of our *R. ventralis* genome assembly, we also performed a chromosomal collinearity analysis between *R. ventralis* and its relative *R. nasutus* [39] by using annotated protein-coding sequences and gene structures with JCVI v190213 [40]. MCscanX v0.8 [41] was employed to identify collinear regions of the 25 chromosomes of *R. ventralis*. TBtools-II v2.310 [42] was applied to visualize genomic features in a circos plot.

### 2.6. Genome-Wide Identification of AMP Sequences for Localization of AMP-Containing Genes

To predict putative AMPs, we downloaded 14,957 known AMP sequences from four public databases (accessed date: 13 January 2025), including Database of Antimicrobial Activity and Structure of Peptides (DBAASP, https://dbaasp.org/about, accessed date: 13 January 2025), Antimicrobial Peptide Database (APD3, https://aps.unmc.edu/AP/, accessed date: 13 January 2025), Data bank antimicrobial peptides (dbAMP, https://awi.cuhk.edu.cn/dbAMP/, accessed date: 13 January 2025) and Data repository of antimicrobial peptides (DRAMP, http://dramp.cpu-bioinfor.org/, accessed date: 13 January 2025). We constructed a reference database using the downloaded sequences with formatdb software v2.2.26. Subsequently, the protein sequences of *R. ventralis* and *R. nasutus* were aligned against this database using TBLASTN v 2.2.26 (-word_size 7-evalue 1 × 10^−5^-outfmt 6). Only BLAST hits exhibiting a query alignment ratio exceeding 0.8 and an query coverage of over 0.6 were retained [15]. Finally, putative AMPs were extracted from their corresponding peptide sequences using a custom script based on this refined database. MG2C v2.1 (http://mg2c.iask.in/mg2c_v2.1/, accessed on 25 July 2025) was employed to map putative AMPs onto the *R. ventralis* chromosomes, with the top ten AMP-containing gene types (Table 2) displayed in distinct color gradients. Phylogenetic analysis was performed on sequences from the major AMP-containing gene category. Multiple sequence alignment was first conducted using MUSCLE v3.8.31 [43], followed by the construction of a maximum likelihood phylogenetic tree with MEGA-X [44], both using default parameters. The phylogenetic tree was further refined for optimal visual presentation utilizing the Interactive Tree of Life (iTOL) v7 platform (https://itol.embl.de/).

## 3. Results

### 3.1. Summary of the Genome Sequencing Data and Assembly

MGI, PacBio HiFi and Hi-C libraries were generated with 54.75, 25.76 and 106.45 Gb of sequencing reads, respectively; for the transcriptome sequencing, approximately 6~9 Gb of clean data from each tissue were obtained (see more details in Table 1). The genome size of *R. ventralis* was estimated to be 1.01 Gb using the 17-mer frequency distribution analysis (Table 2; Figure 1b).

The assembly of HiFi long reads covered a 1.03-Gb genome with a contig N50 of 25.91 Mb. Using Hi-C scaffolding, the final genome assembly of *R. ventralis* was 1015.9 Mb in length, within them 987.4 Mb (97.19%) were successfully anchored onto 25 chromosomes (Table 2; Figure 1d). Assessment of genome completeness revealed (1) CRAQ R-AQI (95.47) and S-AQI (99.70) scores, and (2) Merqury QV scores of 49.213 (MGI short reads) and 64.056 (HiFi long reads) and BUSCO completeness (99.72%), validating the high quality of this chromosome-level assembly. See detailed statistics of the assembly and the 25 chromosomes (Chr 1 to 25) in Table 2 and Table 3, respectively.

### 3.2. Genome Prediction and Annotation

Integrated homology-based and *de novo* analyses revealed that repetitive elements comprise 51.00% (545.4 Mb) of the assembled genome (Table 2, Figure 1c). Among the repeat elements, DNA transposons, long interspersed nuclear elements (LINEs), short interspersed nuclear elements (SINEs), and long terminal repeats (LTRs) accounted for 27.81%, 3.41%, 0.52%, and 8.04%, respectively. We employed three different methods (including *de novo*, homology and transcriptome-based) to predict protein-coding genes. In total, 23,220 genes were annotated in the *R. ventralis* genome assembly. Subsequent function assignment of these genes against five public databases was conducted. A total of 23,171 genes, accounting for 99.79% of the predicted genes were functionally annotated (see more details in Table 4). Notably, 94.07% completeness of the predicted protein-coding genes was exhibited in the genome assembly. Genomic features including chromosome numbers, gene distribution, GC content, repeat elements, and collinear blocks were illustrated in Figure 1c.

### 3.3. Genome Synteny

**Figure 2:**
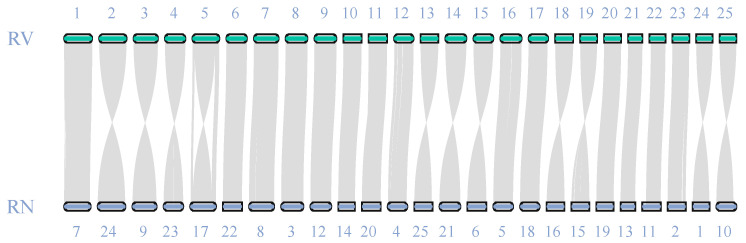
Genome synteny between *R. ventralis* (RV) and its relative *R. nasutus* (RN) [39].

Chromosomal collinearity analysis revealed strong genomic synteny between the *R. ventralis* (RV) and *R. nasutus* (RN) genomes (Figure 2). All the 25 chromosomes displayed one-to-one synteny between the two relative species, validating the high quality and completeness of our *R. ventralis* assembly (Figure 1c) established in this study.

### 3.4. Identification and Localization of AMP-Containing Genes

Genome-wide screening revealed 561 putative AMP-containing genes in *R. ventralis* (Table S1); among them 524 (93.4%) were functionally annotated with classification into 185 distinct categories through database comparisons. The remaining 37 unannotated ones were mapped to *R. ventralis* genes with a prediction to encode 23 histone subunits, 4 complement system components, 2 lysozymes, and 8 other proteins (including RV_ACBP, RV_Ugt2a1, RV_KALM, RV_KPYM, RV_NLRP1 and RV_RNSL3).

**Figure 3:**
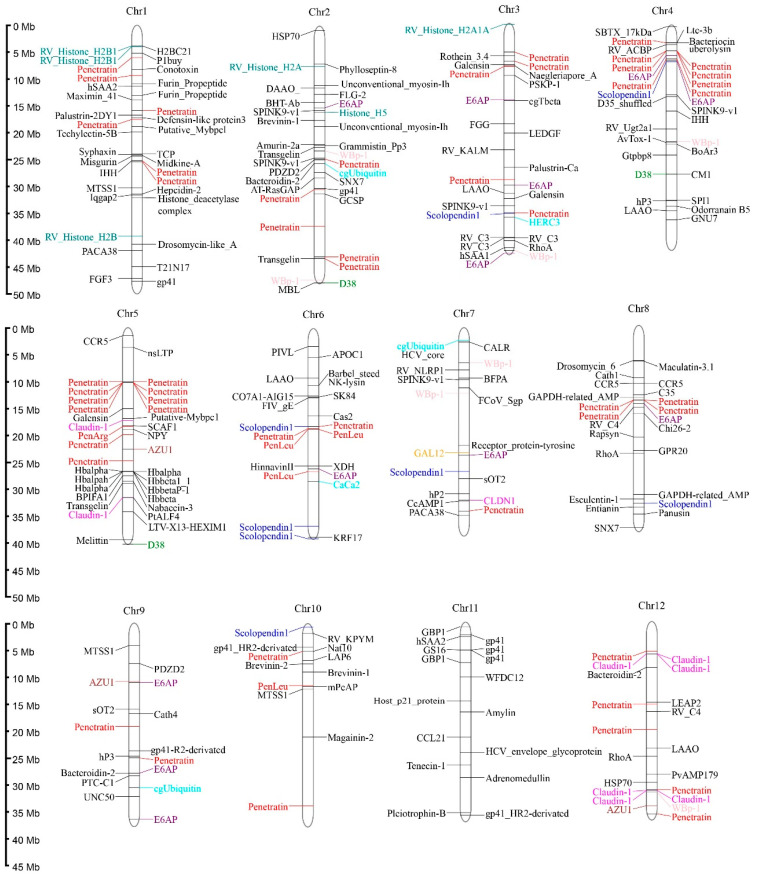

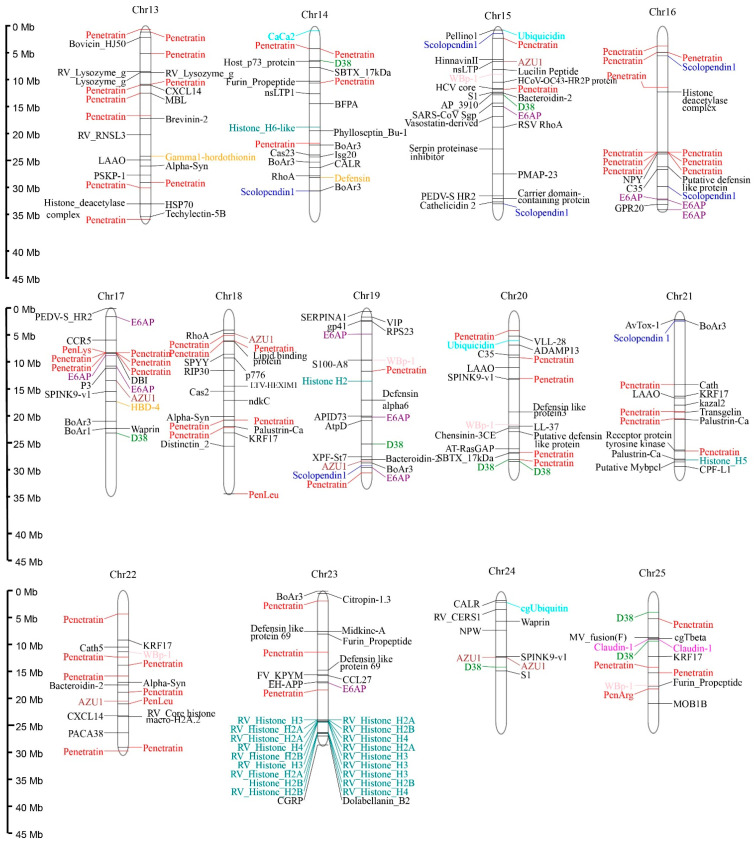
Distribution of 561 AMP-containing genes on the 25 chromosomes of the *R. ventralis* genome. Various colors for different AMP categories: Penetratin, red; Histone, blue-green; E6AP, purple; Scolopendin 1, blue; D38, green; WBp-1, pink; Defensin, orange; Claudin 1, magenta; AZU1, brown; Ubiquitin, cyan; the others, black.

Predicted AMPs were widely distributed across the 25 chromosomes of *R. ventralis*, with Chr 1–4 and 23 each containing ≥30 AMP-containing genes. Specifically, the Chr 24 exhibited the lowest AMP density with only 10 AMP-containing genes. These genes exhibited diverse classifications, with the ten most abundant categories (Table 5) including Penetratin (and deviated analogs, 21.74%,), Histone (5.70%), E6AP (4.09%), Scolopendin 1 (2.67%), D38 (2.31%), WBp-1 (2.13%), Defensin (2.13%), Claudin 1 (1.96%), Azurocidin (AZU1, 1.78%), and Ubiquitin (1.60%). Notably, penetratin genes formed distinct clusters on the Chr 4–5 and 16–17; histone and hemoglobin AMP-containing genes showed clustering on the Chr 5 and 23, respectively (see Figure 3). A phylogenetic analysis resolved the 122 penetratin-containing genes into several major clusters, with the most abundant one being identified as the *Hox* family (33 genes; see more details in Figure S1).

*R. nasutus*, a close relative of *R. ventralis*, is another endangered endemic fish restricted to the Yellow River. To compare their AMP profiles of these two phylogenetically related but geographically isolated species, we performed a genome-wide AMP-containing gene screening in *R. nasutus* [39] as well. Obviously, this species also possesses an extensive repertoire of putative AMP-containing genes (a total of 855; see Table S1). Among them, 24 annotated sequences were mapped to the histone-family members. Detailed numbers of the top ten categories of AMP-containing genes identified in both *R. ventralis* and *R. nasutus* are listed (in Table 5) for comparison.

## 4. Discussion

As an endangered and endemic cyprinid species of the Yangtze River, *R. ventralis* plays important roles with regard to biodiversity, adaptation, and population restoration. Despite its considerable ecological and economic importance, *R. ventralis* remains poorly studied. In this study, we produced a high-quality chromosome-level genome assembly for *R. ventralis*, with 97.19% (987.4 Mb) of the entire sequences anchored onto 25 chromosomes, consistent with the known karyotype for this species. To validate the quality of this genome assembly, we also performed chromosomal collinearity analysis with that of its close relative *R. nasutus*, which possesses a complete telomere-to-telomere reference genome assembly [39]. The high degree of synteny observed across the total 25 chromosome pairs (Figure 2) confirmed the high quality of our *R. ventralis* genome assembly (Figure 1c), which will be beneficial for further evolutionary and functional as well as conservative studies for this endangered species.

As part of the innate immune system, AMPs always play a critical role in immune defense and immune regulation. According to the genome-wide identification of AMPs, the entire number and category of AMPs are various in different species. The substantial difference in putative AMP-containing genes between *R. ventralis* (561) and *R. nasutus* (855) reveals different AMP profiles, which might be due to their distinct surrounding microbial environments in their respective riverine ecosystems. In contrast to prior studies that relied on a single AMP database, our present investigation integrated the most recent data from four public databases to ensure comprehensive coverage. Furthermore, we implemented strict e-value thresholds during sequence alignment to enhance detection reliability. In our current study, the Penetratin family (including its synthetic analogs) were identified as the predominant class of AMP-containing genes in both *R. ventralis* and *R. nasutus* genomes. Previous studies have demonstrated that thrombin are most abundant across 27 species, such as amphibious mudskippers [16], the lined seahorse (*Hippocampus erectus*) [15], the giant grouper (*Epinephelus lanceolatus*) [14], tilapias (*Oreochromis niloticus* and *O. aureus*), black rockfish (*Sebastes schlegelii*) [17] and golden pompano (*Trachinotus ovatus*) [45]. These differences may be caused by the distinct inhabited environments of these fish species, since the two species investigated in this study are living in freshwater ecosystems but the other examined fish species are commonly resident in seawater or euryhaline ecosystems.

Penetratin was initially discovered in the Antennapedia homeodomain of *Drosophila*, and belong to the cell-penetrating peptide (CPP) family [46]. Both penetratin and its derived analogs exhibit dual functional advantages, e.g., membrane-penetrating capability coupled with antimicrobial activity against bacteria and fungi [47,48,49,50]. Compared to native penetratin, a synthetic analog penArg was demonstrated to have enhanced antibacterial activity against *Staphylococcus aureus* and *Escherichia coli*, while PenLys and PenLeu were exhibited to possess significantly reduced cytotoxicity [48]. The high abundance of penetratin indicate its potential immune defense against both bacterial and fungi pathogens in *Rhinogobio* species. The second top abundant AMP-containing gene category in both *Rhinogobio* species was Histone. In addition to conventional antibacterial peptides, Histone-derived AMPs can synergize with other AMPs (e.g., magainin-2 and LL-37) to enhance antimicrobial activity, thus inducing the expression of major histocompatibility complex (MHC)-related genes [51]. We also identified several novel potential AMP-containing gene categories in both *Rhinogobio* fishes, such as antiviral CRISPR-associated endoribonuclease Cas2 family [52], intestinal microbiota-regulated bacteroidin-2 [53], and (3) immunomodulatory azurocidin 1 (enhancing cytokine release) [54], which are worthy of more investigations. In brief, our present study identified differential AMP-containing genes in a high through-put way between two *Rhinogobio* species that originated from different riverine systems.

## 5. Conclusions

In this study, we assembled a high-quality chromosome-level genome for the endangered endemic species *R. ventralis* through an integrative approach of MGI short-read, PacBio long-read, and Hi-C sequencing technologies. The final genome assembly was 1015.9 Mb with high continuity and completeness. Comparative synteny analysis of the 25 chromosomal pairs between *R. ventralis* (RV) and its relative *R. nasutus* (RN) revealed conserved one-to-one correspondence. To obtain comprehensive AMPs of *Rhinogobio* species from distinct riverine ecosystems, we performed genome-wide prediction in both *Rhinogobio* fishes to identify 561 and 855 putative AMP-containing genes, respectively. Among them, Penetratin and Histone genes represented the most abundant categories in both species. In *R. ventralis*, the identified AMP-containing genes were widely distributed across the total 25 chromosomes, with five chromosomes harboring ≥30 genes each. The high-quality genome assembly and comprehensive AMP-containing gene characterization of *R. ventralis* establish a potentially valuable genomic resource that could inform conservation strategies for this endangered species, elucidate mechanisms of lotic adaptation, and facilitate development of novel antimicrobial agents or drug delivery systems for both aquaculture and human medicine applications.

## Figures and Tables

**Table 1:** Summary of genome and transcriptome sequencing data from an adult female *R. ventralis*. * For the PacBio HiFi sequencing, this number is read N50; for others, it denotes read length.

Sequencing	Library Type		Raw Data (Gb)	Clean Data (Gb)	Read N50/Length (bp)	Coverage (×)
Whole-genome sequencing	MGI		55.78	30.78	150	30.78
	PacBio-HiFi		-	42.53	15,765 *	41.90
	Hi-C		106.45	95.77	150	94.35
Transcriptome sequencing	RNA	Brain	9.24	7.94	150	
		Eye	8.25	7.42	150	
		Gill	7.69	6.84	150	
		Muscle	6.68	6.02	150	
		Heart	6.69	6.08	150	
		Intestine	7.98	6.78	150	
		Kidney	6.29	5.67	150	
		Liver	7.72	6.91	150	
		Ovary	10.01	8.97	150	

**Table 2:** Statistic of the assembled *R. ventrali* genome.

Category	Data
Genome survey (Gb)	1.01
Genome length (bp)	1,015,928,399
Longest scaffold (bp)	54,161,874
Number of scaffolds	48
Contig N50	25.91
Scaffold N50	39.99
GC content	38.8%
CRAQ score	R-AQI = 95.47, S-AQI = 99.70
Merqury QV score	49.213 (short-read NGS), 64.056 (HiFi long-reads)
Completeness score	S: 99.42%, D: 0.30%, F: 0.14%, I: 0.00%, M: 0.14%
Anchor ratio	97.19%
Number of chromosomes	25
Chromosome length (bp)	987,426,077
Repetitive sequence	51.00%

**Table 3:** Length of the assembled chromosomes in the final genome assembly.

Chromosome No.	Length (bp)	Chromosome No.	Length (bp)
Chr 1	54,161,874	Chr 14	39,366,162
Chr 2	51,912,737	Chr 15	38,936,029
Chr 3	46,659,604	Chr 16	38,319,520
Chr 4	45,454,307	Chr 17	37,929,373
Chr 5	43,671,396	Chr 18	37,250,726
Chr 6	42,439,934	Chr 19	36,383,716
Chr 7	41,984,274	Chr 20	34,013,917
Chr 8	41,542,622	Chr 21	33,990,390
Chr 9	41,012,051	Chr 22	33,137,678
Chr 10	40,964,245	Chr 23	31,155,457
Chr 11	40,208,822	Chr 24	28,609,039
Chr 12	39,998,020	Chr 25	28,453,069
Chr 13	39,871,115		

**Table 4:** Function annotation of the total protein-coding genes. Total represents the number of annotated genes with at least one hit from the five searched public databases.

Category	Number	Percentage (%)
Total	23,171	99.79
Swissprot	18,889	81.35
KEGG	14,759	63.56
KOG	13,592	58.54
GO	14,078	60.63
NCBI NR	23,168	99.78
Completeness	3424	94.07

**Table 5:** Number of the top ten categories of AMP-containning genes in *R. ventralis* and *R. nasutus*.

Category	*R. ventralis*	*R. nasutus*
Penetratin	122	166
Histone	32	154
E6AP	23	27
Scolopendin 1	15	15
D38	13	17
WBp-1	12	15
Defensin	12	14
Claudin 1	10	25
AZU1	10	13
Ubiquitin	9	7

## Data Availability

The original contributions presented in the study are included in the article. Further inquiries can be directed to the corresponding authors.

## Supplementary Materials

The following supporting information can be downloaded at: https://www.mdpi.com/article/10.3390/biology14111486/s1. Figure S1: Phylogenetic analysis of penetratin-containing genes in *R. ventralis*. Table S1: A genome-wide comparison of AMP-containing genes between *R. ventralis* and its relative *R. nasutus*.

## References

[1] Gao X., Zeng Y., Wang J.W., Liu H.Z. (2010). Immediate impacts of the second impoundment on fish communities in the Three Gorges Reservoir. Environ. Biol. Fish..

[2] Li Y.L., Yang J.J., Wang Y.H., Wu H.C., Ma Y.M., Wu F.X. (2025). Sediment eDNA reveals damming triggered changes in algal and fish communities at the Three Gorges Reservoir in China. Environ. Res..

[3] Yang H.L., Shen L., He Y.F., Tian H.W., Gao L., Wu J.M., Mei Z.G., Wei N., Lin W., Zhu T.B. (2024). Status of aquatic organisms resources and their environments in Yangtze River system (2017–2021). Aquac. Fish..

[4] Gao X., Masami F., Winemiller K.O., Lin P.C., Li M.Z., Liu H.Z. (2019). Regime shift in fish assemblage structure in the Yangtze River following construction of the Three Gorges Dam. Sci. Rep..

[5] Yue P.Q., Chen Y.Y. (1998). Gobioninae. Fauna Sinica: Osteichthys, Cypriniformes.

[6] Wang X.Z., Liu H.Z. (2005). Phylogenetic relationships of the Chinese cyprinid genus Rhinogobio Bleeker (Teleostei: Cyprinidae) based on sequences of the mitochondrial DNA control region, with comments on character adaptations. Hydrobiologia.

[7] Liu F., Wang J., Cao W. (2013). Long-term changes in fish assemblage following the impoundments of the Three Gorges Reservoir in Hejiang, a protected reach of the upper Yangtze River. Knowl. Manag. Aquat. Ecosyst..

[8] Liu F., Wang J.W., Liu H.Z. (2019). Seasonal variations in food resource partitioning among four sympatric gudgeon species in the upper Yangtze River. Ecol. Evol..

[9] Wu X., Cheng B., Xue M., Jiang N., Li X., Hu X., Li X., Zhu T., Zhu Y., Zhou Y. (2024). Isolation, Characterization, and Pathogenicity of an *Aeromonas veronii* Strain Causing Disease in *Rhinogobio ventralis*. Fishes.

[10] Huang K., Wang R., Hu G., Zhou W., Li W., Zou H., Wang G., Li M. (2024). Immune response of *Rhinogobio ventralis* to *Ichthyophthirius multifiliis* infection: Insights from histopathological and real-time gene expression analyses. Fish Shellfish Immunol..

[11] Li H., Niu J., Wang X., Niu M., Liao C. (2023). The Contribution of Antimicrobial Peptides to Immune Cell Function: A Review of Recent Advances. Pharmaceutics.

[12] Masso-Silva J.A., Diamond G. (2014). Antimicrobial peptides from fish. Pharmaceuticals.

[13] Xiao X., Lu H., Zhu W., Zhang Y., Huo X., Yang C., Xiao S., Zhang Y., Su J.A. (2022). Novel Antimicrobial Peptide Derived from Bony Fish IFN1 Exerts Potent Antimicrobial and Anti-Inflammatory Activity in Mammals. Microbiol. Spectr..

[14] Wang D., Chen X., Zhang X., Li J., Yi Y., Bian C., Shi Q., Lin H., Li S., Zhang Y. (2019). Whole Genome Sequencing of the Giant Grouper (*Epinephelus lanceolatus*) and High-Throughput Screening of Putative Antimicrobial Peptide Genes. Mar. Drugs.

[15] Chen X., Yi Y., You X., Liu J., Shi Q. (2019). High-Throughput Identification of Putative Antimicrobial Peptides from Multi-Omics Data of the Lined Seahorse (*Hippocampus erectus*). Mar. Drugs.

[16] Yi Y., You X., Bian C., Chen S., Lv Z., Qiu L., Shi Q. (2017). High-Throughput Identification of Antimicrobial Peptides from Amphibious Mudskippers. Mar. Drugs.

[17] Zhang M., Cao M., Xiu Y., Fu Q., Yang N., Su B., Li C. (2021). Identification of Antimicrobial Peptide Genes in Black Rockfish *Sebastes schlegelii* and Their Responsive Mechanisms to *Edwardsiella tarda* Infection. Biology.

[18] Wen Z., Wei X., Chen J., Li Y., Zhou B., Zhang C., Fu P., Prathomya P., Li R., Lv Y. (2024). Chromosome-level genome assemblies of vulnerable male and female elongate loach (*Leptobotia elongata*). Sci. Data.

[19] He C., Zhang X., Wen Z., Shi Q., Song Z. (2024). A chromosome-scale reference genome assembly for *Triplophysa lixianensis*. Sci. Data.

[20] Lv Y., Li Y., Huang Y., Wang J., Tian Z., He Y., Shi J., Huang Z., Wen Z., Shi Q. (2024). Deciphering genome-wide molecular pathways for exogenous Aeromonas hydrophila infection in wide-bodied sand loach (*Sinibotia reevesae*). Aquac. Rep..

[21] Vurture G.W., Sedlazeck F.J., Nattestad M., Underwood C.J., Fang H., Gurtowski J., Schatz M.C. (2017). GenomeScope: Fast reference-free genome profiling from short reads. Bioinformatics.

[22] Liu B., Shi Y., Yuan J., Hu X., Zhang H., Li N., Li Z., Chen Y., Mu D., Wei F. (2013). Estimation of genomic characteristics by analyzing k-mer frequency in *de novo* genome projects. Quant. Biol..

[23] Cheng H., Concepcion G.T., Feng X., Zhang H., Li H. (2021). Haplotype-resolved *de novo* assembly using phased assembly graphs with hifiasm. Nat. Methods.

[24] Langmead B., Salzberg S.L. (2012). Fast gapped-read alignment with Bowtie 2. Nat. Methods.

[25] Varoquaux N., Lajoie B.R., Viara E., Chen C., Vert J.P., Heard E., Job Dekker J., Barillot E. (2015). HiC-Pro: An optimized and flexible pipeline for Hi-C data processing. Genome Biol..

[26] Zhou C., McCarthy S.A., Durbin R. (2023). YaHS: Yet another Hi-C scaffolding tool. Bioinformatics.

[27] Durand N.C., Robinson J.T., Shamim M.S., Machol I., Mesirov J.P., Lander E.S., Aiden E.L. (2016). Juicebox Provides a Visualization System for Hi-C Contact Maps with Unlimited Zoom. Cell Syst..

[28] Xu M., Guo L., Gu S., Wang O., Zhang R., Peters B.A., Fan G., Liu X., Xu X., Deng L. (2020). TGS-GapCloser: A fast and accurate gap closer for large genomes with low coverage of error-prone long reads. Gigascience.

[29] Li K., Xu P., Wang J., Yi X., Jiao Y. (2023). Identification of errors in draft genome assemblies at single-nucleotide resolution for quality assessment and improvement. Nat. Commun..

[30] Rhie A., Walenz B.P., Koren S., Phillippy A.M. (2020). Merqury: Reference-free quality, completeness, and phasing assessment for genome assemblies. Genome Biol..

[31] Huang N., Li H. (2023). compleasm: A faster and more accurate reimplementation of BUSCO. Bioinformatics.

[32] Tarailo-Graovac M., Chen N. (2009). Using Repeat Masker to identify repetitive elements in genomic sequences. Curr. Protoc. Bioinform..

[33] Xu Z., Wang H. (2007). LTR_FINDER: An efficient tool for the prediction of full-length LTR retrotransposons. Nucleic Acids Res..

[34] Flynn J.M., Hubley R., Goubert C., Rosen J., Clark A.G., Feschotte C., Smit A.F. (2020). RepeatModeler2 for automated genomic discovery of transposable element families. Proc. Natl. Acad. Sci. USA.

[35] Stanke M., Keller O., Gunduz I., Hayes A., Waack S., Morgenstern B. (2006). AUGUSTUS: Ab initio prediction of alternative transcripts. Nucleic Acids Res..

[36] Keilwagen J., Hartung F., Grau J. (2019). GeMoMa: Homology-based gene prediction utilizing intron position conservation and RNA-seq data. Gene Prediction.

[37] Haas B.J., Papanicolaou A., Yassour M., Grabherr M., Blood P.D., Bowden J., Couger M.B., Eccles D., Li B., Lieber M. (2013). De novo transcript sequence reconstruction from RNA-seq using the Trinity platform for reference generation and analysis. Nat. Protoc..

[38] Haas B.J., Salzberg S.L., Zhu W., Pertea M., Allen J.E., Orvis J., White O., Buell C.R., Wortman J.R. (2008). Automated eukaryotic gene structure annotation using EVidenceModeler and the Program to Assemble Spliced Alignments. Genome Biol..

[39] Jiang C., Du Y., Lou Z., Zhang Y., Wang T. (2025). Telomere-to-telomere reference genome of *Rhinogobio nasutus*, an endangered endemic fish from the Yellow River. Sci. Data.

[40] Tang H., Krishnakumar V., Bidwell S., Rosen B., Chan A., Zhou S., Gentzbittel L., Childs K.L., Yandell M., Gundlach H. (2014). An improved genome release (version Mt4.0) for the model legume Medicago truncatula. BMC Genom..

[41] Wang Y., Tang H., Debarry J.D., Tan X., Li J., Wang X., Lee T.H., Jin H., Marler B., Guo H. (2012). MCScanX: A toolkit for detection and evolutionary analysis of gene synteny and collinearity. Nucleic Acids Res..

[42] Chen C., Wu Y., Li J., Wang X., Zeng Z., Xu J., Liu Y., Feng J., Chen H., He Y. (2023). TBtools-II: A “one for all, all for one” bioinformatics platform for biological big-data mining. Mol. Plant.

[43] Edgar R.C. (2010). Quality measures for protein alignment benchmarks. Nucleic Acids Res..

[44] Kumar S., Stecher G., Li M., Knyaz C., Tamura K. (2018). MEGA X: Molecular Evolutionary Genetics Analysis across Computing Platforms. Mol. Biol. Evol..

[45] Liang Y., Pan J.M., Zhu K.C., Xian L., Guo H.Y., Liu B.S., Zhang N., Yang J.W., Zhang D.C. (2023). Genome-Wide Identification of Trachinotus ovatus Antimicrobial Peptides and Their Immune Response against Two Pathogen Challenges. Mar. Drugs.

[46] Derossi D., Joliot A.H., Chassaing G., Prochiantz A. (1994). The third helix of the Antennapedia homeodomain translocates through biological membranes. J. Biol. Chem..

[47] Garibotto F.M., Garro A.D., Rodríguez A.M., Raimondi M., Zacchino S.A., Perczel A., Somlai C., Penke B., Enriz R.D. (2011). Penetratin analogues acting as antifungal agents. Eur. J. Med. Chem..

[48] Bahnsen J.S., Franzyk H., Sandberg-Schaal A., Nielsen H.M. (2013). Antimicrobial and cell-penetrating properties of penetratin analogs: Effect of sequence and secondary structure. Biochim. Biophys. Acta.

[49] Zhu W.L., Lan H., Park I.S., Kim J.I., Jin H.Z., Hahm K.S., Shin S.Y. (2006). Design and mechanism of action of a novel bacteria-selective antimicrobial peptide from the cell-penetrating peptide Pep-1. Biochem. Biophys. Res. Commun..

[50] Zhu W.L., Shin S.Y. (2009). Antimicrobial and cytolytic activities and plausible mode of bactericidal action of the cell penetrating peptide penetratin and its lys-linked two-stranded peptide. Chem. Biol. Drug Des..

[51] Duong L., Gross S.P., Siryaporn A. (2020). A novel antibacterial strategy: Histone and antimicrobial peptide synergy. Microb. Cell.

[52] Beloglazova N., Brown G., Zimmerman M.D., Proudfoot M., Makarova K.S., Kudritska M., Kochinyan S., Wang S., Chruszcz M., Minor W. (2008). A novel family of sequence-specific endoribonucleases associated with the clustered regularly interspaced short palindromic repeats. J. Biol. Chem..

[53] Torres M.D.T., Brooks E.F., Cesaro A., Sberro H., Gill M.O., Nicolaou C., Bhatt A.S., de la Fuente-Nunez C. (2024). Mining human microbiomes reveals an untapped source of peptide antibiotics. Cell.

[54] Soehnlein O., Lindbom L. (2009). Neutrophil-derived azurocidin alarms the immune system. J. Leukoc. Biol..

