# Supplementary material for: Chromosome-Scale Genome Assembly and Genome-Wide Identification of Antimicrobial Peptide-Containing Genes in the Endangered Long-Finned Gudgeon Fish (Rhinogobio ventralis)
Source: Biology (Basel). 2025 Oct 24;14(11):1486. doi: 10.3390/biology14111486 (PMC12649986; doi:10.3390/biology14111486)
Supplement: Supplementary file 1 [file biology-14-01486-s001.zip › biology-3868709-supplementary.pdf]

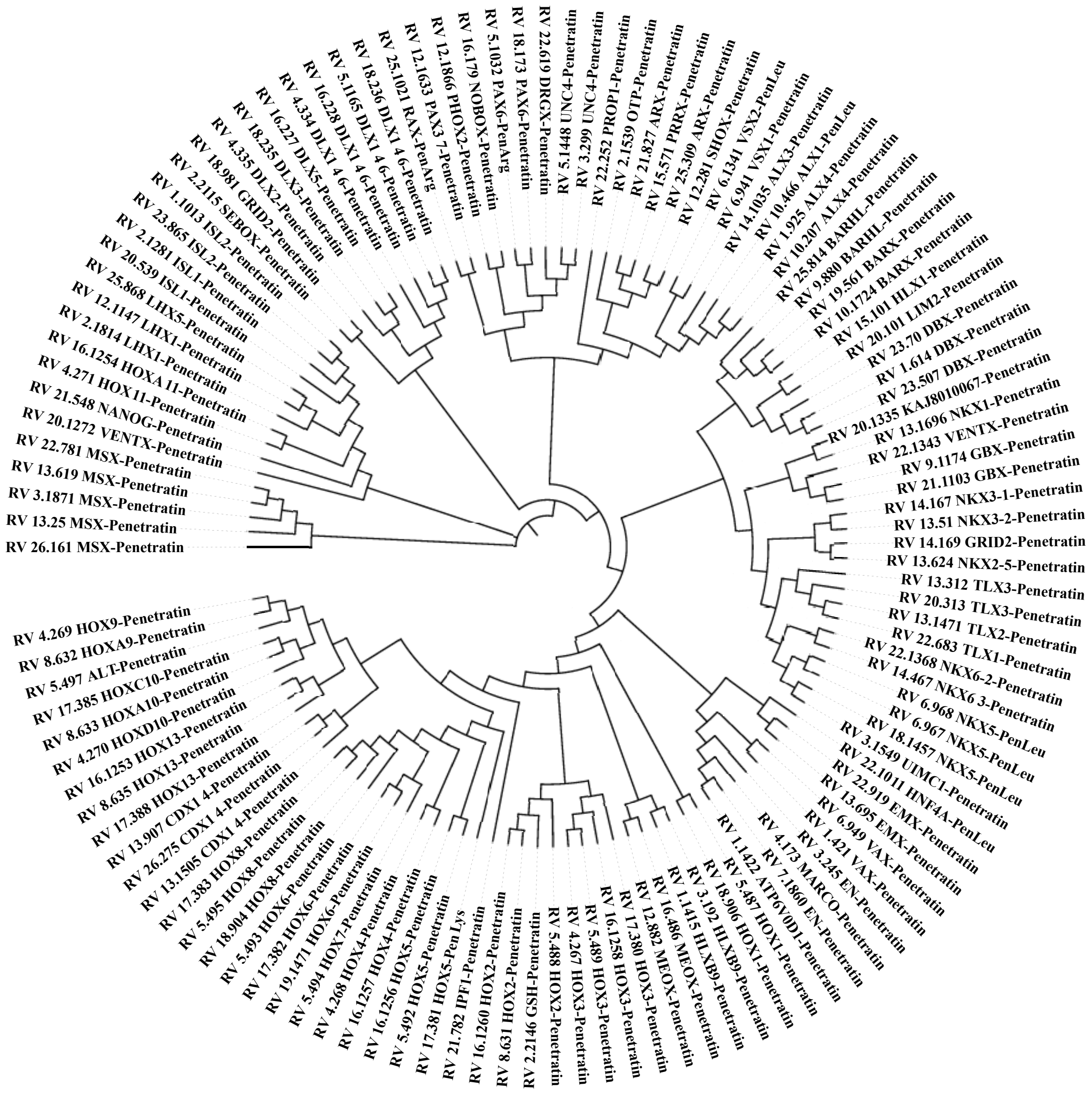

Supplementary Figure S1. Phylogenetic analysis of penetratin-containing genes in *R. ventralis*. The tree was constructed from genome-wide protein sequences aligned with MUSCLE v3.8.31. A maximum-likelihood phylogeny was generated using MEGA-X with 1,000 bootstrap replicates. Individual branch is labeled with each gene ID and associated AMP name.
